# Supplementary material for: Quantity and quality of minichromosome maintenance protein complexes couple replication licensing to genome integrity
Source: Commun Biol. 2024 Feb 9;7:167. doi: 10.1038/s42003-024-05855-w (PMC10858283; doi:10.1038/s42003-024-05855-w)
Supplement: Supplementary file 2 — Reporting Summary [file 42003_2024_5855_MOESM2_ESM.pdf]

nature portfolio

Corresponding author(s): Hana Polasek-Sedlackova

Last updated by author(s): Jan 5, 2024

Reporting Summary

Nature Portfolio wishes to improve the reproducibility of the work that we publish. This form provides structure for consistency and transparency in reporting. For further information on Nature Portfolio policies, see our Editorial Policies and the Editorial Policy Checklist.

Statistics

For all statistical analyses, confirm that the following items are present in the figure legend, table legend, main text, or Methods section.

n/a	Confirmed	
☒	☐	The exact sample size (*n*) for each experimental group/condition, given as a discrete number and unit of measurement
☒	☐	A statement on whether measurements were taken from distinct samples or whether the same sample was measured repeatedly
☒	☐	The statistical test(s) used AND whether they are one- or two-sided<br>*Only common tests should be described solely by name; describe more complex techniques in the Methods section.*
☒	☐	A description of all covariates tested
☒	☐	A description of any assumptions or corrections, such as tests of normality and adjustment for multiple comparisons
☒	☐	A full description of the statistical parameters including central tendency (e.g. means) or other basic estimates (e.g. regression coefficient) AND variation (e.g. standard deviation) or associated estimates of uncertainty (e.g. confidence intervals)
☒	☐	For null hypothesis testing, the test statistic (e.g. *F*, *t*, *r*) with confidence intervals, effect sizes, degrees of freedom and *P* value noted<br>*Give P values as exact values whenever suitable.*
☒	☐	For Bayesian analysis, information on the choice of priors and Markov chain Monte Carlo settings
☒	☐	For hierarchical and complex designs, identification of the appropriate level for tests and full reporting of outcomes
☒	☐	Estimates of effect sizes (e.g. Cohen's *d*, Pearson's *r*), indicating how they were calculated

Our web collection on statistics for biologists contains articles on many of the points above.

Software and code

Policy information about availability of computer code

Data collection	ZEN black acquistion software (ZEISS, version 2.3) and LSM880-Airyscan confocal microscope was used to generate a representative image in Figure 1c.
Data analysis	ImageJ software (FIJI, 1.53s) was used to prepare a representative image in Figure 1c.

For manuscripts utilizing custom algorithms or software that are central to the research but not yet described in published literature, software must be made available to editors and reviewers. We strongly encourage code deposition in a community repository (e.g. GitHub). See the Nature Portfolio guidelines for submitting code & software for further information.

Data

Policy information about availability of data

All manuscripts must include a data availability statement. This statement should provide the following information, where applicable:

- Accession codes, unique identifiers, or web links for publicly available datasets
- A description of any restrictions on data availability
- For clinical datasets or third party data, please ensure that the statement adheres to our policy

Our paper contains only published literature. The data presented in Table 1 were extracted from reference 40 based on mass spectrometry datasets deposited on the ProteomeXchange Consortium under accession numbers PXD002815 [https://proteomecentral.proteomexchange.org/cgi/GetDataset?ID=PXD002815] and PXD002829 [https://proteomecentral.proteomexchange.org/cgi/GetDataset?ID=PXD002829].

Research involving human participants, their data, or biological material

Policy information about studies with human participants or human data. See also policy information about sex, gender (identity/presentation), and sexual orientation and race, ethnicity and racism.

Reporting on sex and gender	None
Reporting on race, ethnicity, or other socially relevant groupings	None
Population characteristics	None
Recruitment	None
Ethics oversight	None

Note that full information on the approval of the study protocol must also be provided in the manuscript.

Field-specific reporting

Please select the one below that is the best fit for your research. If you are not sure, read the appropriate sections before making your selection.

☒ Life sciences ☐ Behavioural & social sciences ☐ Ecological, evolutionary & environmental sciences

For a reference copy of the document with all sections, see nature.com/documents/nr-reporting-summary-flat.pdf

Life sciences study design

All studies must disclose on these points even when the disclosure is negative.

Sample size	not relevant
Data exclusions	not relevant
Replication	not relevant
Randomization	not relevant
Blinding	not relevant

Reporting for specific materials, systems and methods

We require information from authors about some types of materials, experimental systems and methods used in many studies. Here, indicate whether each material, system or method listed is relevant to your study. If you are not sure if a list item applies to your research, read the appropriate section before selecting a response.

Materials & experimental systems

n/a	Involved in the study	
☐	☒	Antibodies
☐	☒	Eukaryotic cell lines
☒	☐	Palaeontology and archaeology
☒	☐	Animals and other organisms
☒	☐	Clinical data
☒	☐	Dual use research of concern
☒	☐	Plants

Methods

n/a	Involved in the study	
☒	☐	ChIP-seq
☒	☐	Flow cytometry
☒	☐	MRI-based neuroimaging

Antibodies

Antibodies used	Primary antibodies to generate a representative immunofluorescence image in Figure 1c using confocal microscopy were used as follows: MCM2 (mouse, Novus Biologicals, H00004171-M01, clone 6A8, 1:1,000) and TIMELESS (rabbit, Abcam, ab109512, clone EPR5275, 1:500). Secondary antibody conjugates were goat anti-mouse Alexa Fluor 488 (A11029, Thermo Fischer Scientific), and goat anti-rabbit Alexa Fluor 568 (A21245, Thermo Fischer Scientific).

Validation	MCM2 (mouse, Novus Biologicals, H00004171-M01, clone 6A8, 1:1,000) was previously validated for immunofluorescence experiments in Sedlackova et al., Nature, 2020 Oct 21; 587, 297-302.<br><br>TIMELESS (rabbit, Abcam, ab109512, clone EPR5275, 1:500) was previously validated for immunofluorescence experiments in Somyajit et al., Science 2017 Nov 10;358(6364):797-802.

Eukaryotic cell lines

Policy information about cell lines and Sex and Gender in Research

Cell line source(s)	U2OS cell line (ATCC HTB-96).
Authentication	The U2OS cell line was authenticated by STR profiling.
Mycoplasma contamination	U2OS cell line was routinely tested for mycoplasma (MycoAlert, Lonza) and always found negative.
Commonly misidentified lines (See ICLAC register)	No commonly misidentified cell lines were used in this study.

Plants

Seed stocks	None
Novel plant genotypes	None
Authentication	None
